# Artificial Intelligence Identified Resilient and Vulnerable Female Rats After Traumatic Stress and Ethanol Exposure: Investigation of Neuropeptide Y Pathway Regulation

**DOI:** 10.3389/fnins.2021.772946

**Published:** 2021-12-16

**Authors:** Ray R. Denny, Krista L. Connelly, Marco G. Ghilotti, Joseph J. Meissler, Daohai Yu, Toby K. Eisenstein, Ellen M. Unterwald

**Affiliations:** ^1^Center for Substance Abuse Research, Lewis Katz School of Medicine, Temple University, Philadelphia, PA, United States; ^2^Center for Biostatistics and Epidemiology, Department of Biomedical Education and Data Science, Lewis Katz School of Medicine, Temple University, Philadelphia, PA, United States; ^3^Department of Neural Sciences, Lewis Katz School of Medicine, Temple University, Philadelphia, PA, United States

**Keywords:** anxiety, machine learning, posttraumatic stress disorder (PTSD), alcohol, single prolonged stress (SPS), neuropeptide Y (NPY), extended amygdala, female

## Abstract

Post-traumatic stress disorder (PTSD) is initiated by traumatic-stress exposure and manifests into a collection of symptoms including increased anxiety, sleep disturbances, enhanced response to triggers, and increased sympathetic nervous system arousal. PTSD is highly co-occurring with alcohol use disorder. Only some individuals experiencing traumatic stress develop PTSD and a subset of individuals with PTSD develop co-occurring alcohol use disorder. To investigate the basis of these individual responses to traumatic stress, single prolonged stress (SPS) a rodent model of traumatic stress was applied to young adult female rats. Individual responses to SPS were characterized by measuring anxiety-like behaviors with open field and elevated plus maze tests. Rats were then allowed to drink ethanol under an intermittent two bottle choice procedure for 8 weeks, and ethanol consumption was measured. An artificial intelligence algorithm was built to predict resilient and vulnerable individuals based on data from anxiety testing and ethanol consumption. This model was implemented in a second cohort of rats that underwent SPS without ethanol drinking to identify resilient and vulnerable individuals for further study. Analysis of neuropeptide Y (NPY) levels and expression of its receptors Y1R and Y2R mRNA in the central nucleus of the amygdala (CeA), basolateral amygdala (BLA), and bed nucleus stria terminalis (BNST) were performed. Results demonstrate that resilient rats had higher expression of Y2R mRNA in the CeA compared with vulnerable and control rats and had higher levels of NPY protein in the BNST compared to controls. The results of the study show that an artificial intelligence algorithm can identify individual differences in response to traumatic stress which can be used to predict subsequent ethanol drinking, and the NPY pathway is differentially altered following traumatic stress exposure in resilient and vulnerable populations. Understanding neurochemical alterations following traumatic-stress exposure is critical in developing prevention strategies for the vulnerable phenotype and will help further development of novel therapeutic approaches for individuals suffering from PTSD and at risk for alcohol use disorder.

## Introduction

Post-traumatic stress disorder (PTSD) is a debilitating condition initiated by traumatic stress exposure and is marked by a constellation of symptoms including indelible memories and heightened sympathetic nervous system arousal (Atwoli et al., 2015). In the United States 60% of males and 50% of females are exposed to at least one traumatic stress during their lifetime (Kessler et al., 1995). However, of these individuals only 15–30% go on to develop PTSD (Kessler et al., 1995). While males are exposed to more traumatic stress during life, women have a greater incidence of PTSD (Kessler et al., 1995). There is a 2:1 female to male ratio of individuals with PTSD (Kessler et al., 1995). PTSD is highly co-occurring with other disorders including alcohol use disorder (Kessler et al., 1997), and the rates of co-occurrence vary depending on factors such as sex, age, military/civilian, and location (for review see Gilpin and Weiner, 2017). American veterans diagnosed with PTSD are 3–4.5 times more likely to have a co-occurring AUD (Carter et al., 2011; Seal et al., 2011). PTSD usually precedes the development of an AUD, and when PTSD and AUD present together, individuals have worse outcomes and report more severe symptomologies of both disorders compared to individuals with only PTSD or AUD (Carter et al., 2011; Seal et al., 2011).

Sex differences in the incidence of PTSD and co-occurring AUD exist. Women are more likely to develop an AUD during the same year as they developed PTSD than men (Kessler et al., 1997; McCauley et al., 2012). Women show higher levels of excessive drinking after a traumatic-stress exposure than men (Olff et al., 2007). The average alcohol intake in women with PTSD is significantly correlated with coping motives whereas it is not a significant indicator for men (Lehavot et al., 2014). Sex differences may be present due to differential neurobiological mechanisms (Pineles et al., 2017). Pathways that are affected differently by stressors in males versus females include corticotropin releasing factor (CRF), neuropeptide Y (NPY), glucocorticoid negative feedback, and response to stimuli in the corticolimbic brain region (Rasmusson and Friedman, 2002; Bangasser and Valentino, 2014).

Many useful animal models of PTSD exist, one of which is single prolonged stress (SPS) (Liberzon et al., 1997; Cohen et al., 2011; Zoladz et al., 2012; Enman et al., 2015). SPS recapitulates PTSD symptomology which is marked by increased negative feedback in the hypothalamic-pituitary-adrenal axis (HPA), and increased anxiety and fear behaviors [for review see Daskalakis et al., 2014]. Male and female rats respond differently to SPS (Keller et al., 2015; Pooley et al., 2018; Mancini et al., 2021). Female rats have lower fear retention and enhanced glucocorticoid expression compared to males (Pooley et al., 2018). Unlike male rats, female rats showed enhanced glucocorticoid receptor levels in the dorsal hippocampus and no fear extinction deficits following SPS (Keller et al., 2015). Similar to humans where only a subset of traumatic-stress exposed individuals develop PTSD and co-occurring alcohol abuse, only a subset of rats exposed to stress develop a robust phenotype (Edwards et al., 2013; Manjoch et al., 2016). One objective of this study was to develop a method to identify sub-populations of rats based on behavioral phenotypes that could be used to predict individuals that were vulnerable to the transition from traumatic stress exposure to high ethanol consumption. Artificial intelligence analytical methods were used to achieve this goal.

The second objective of this study was to investigate neurobiological mechanisms that may underlie differences in individual susceptibility to ethanol consumption after exposure to traumatic stress. NPY has been shown to buffer highly stressful stimuli by increasing resiliency to traumatic-stress exposure (Wu et al., 2013). Humans with PTSD have lower levels of NPY compared to controls in both cerebral spinal fluid and plasma (Yehuda et al., 2006; Sah et al., 2009, 2014; Rasmusson et al., 2010). Further, when PTSD goes into remission, NPY levels recover (Yehuda et al., 2006). Importantly, promising results have been obtained from clinical trials suggesting that intranasal NPY can reduce anxiety in persons with PTSD (Sayed et al., 2018). In animal models of traumatic stress, NPY intranasal administration as an early intervention prevents development of PTSD-like symptoms in male rats (Serova et al., 2013; Laukova et al., 2014; Sabban et al., 2015). NPY has also been associated with ethanol consumption. Administration or overexpression of NPY decreases ethanol intake in humans and rodents (Thiele et al., 1998; Badia-Elder et al., 2001; Sparrow et al., 2012; Thorsell and Mathe, 2017). Likewise, ethanol preferring male rats have lower NPY levels in the amygdala, frontal cortex, and hippocampus than non-preferring rats (Ehlers et al., 1998; Hwang et al., 1999). NPY binds to Y1, Y2, Y4, Y5, and Y6 GPCR receptor subtypes with equal binding affinity (Michel et al., 1998). In the central nervous system, Y1R and Y2R are the most predominantly expressed receptors with Y1R located on post-synaptic dendrites and Y2R located on pre-synaptic terminals. As such, Y2R are inhibitory to the release of NPY, glutamate or GABA depending on the cell type. In general, Y1R activation produces anxiolytic effects, whereas Y2R agonists are anxiogenic (for review see Tasan et al., 2016). Y1R and Y2R are found in regions of the amygdala and extended amygdala including the basolateral amygdala (BLA), central nucleus of the amygdala (CeA), and bed nucleus stria terminalis (BNST; Tasan et al., 2016; Wood et al., 2016; Mackay et al., 2019). These regions were chosen for this study because they are critical for fear- and anxiety-related behaviors and ethanol consumption (Hawley et al., 2010; Gilpin et al., 2015; Pleil et al., 2015; Langevin et al., 2016; Young and Tong, 2021).

The goals of this study were to, first, develop a method to reliably forecast which individual rats would consume greater amounts of ethanol following SPS exposure, based on their behavioral phenotype. The second goal of this study was to use this classification to investigate levels of NPY and gene expression its receptors, Y1R and Y2R in brain regions associated with processing fear stimuli, anxiety, and ethanol consumption in rats predicted to be resilient or vulnerable to heightened ethanol consumption following traumatic stress exposure, but prior to exposure to ethanol. As women are twice as likely as men to have PTSD and use ethanol as a significant coping mechanism (Lehavot et al., 2014), female rats were selected for the completion of this study. Results are presented herein that demonstrate that an artificial intelligence algorithm reliably identified individuals based on anxiety-like behaviors after traumatic stress exposure that go on to consume higher or lower amounts of ethanol, and that these populations had differences in NPY in the amygdala and extended amygdala.

## Materials and Methods

### Subjects

Female Sprague-Dawley rats, ordered at 8 weeks of age (Charles River Laboratories, Wilmington, MA, United States), were used in all studies. Rats were allowed to acclimate to the new environment after arriving for 2 days, followed by 4–8 days of minimal handling and daily weighing in preparation for experiments. Rats were housed in a humidity-controlled environment on a 12-h light/dark cycle (lights on at 0700). Animals had continuous access to food and water except during behavioral testing and were housed in pairs with no enrichment devices. All studies were conducted in accordance with the Guide for the Care and Use of Laboratory Animals (National Research Council of the National Academies, 2011). Temple University Institutional Animal Care and Use Committee of Temple University approved all experimental protocols.

### Experiment 1: Phenotypical Analysis of Anxiety-Like Behaviors and Subsequent Ethanol Consumption in Female Rats Exposed to Traumatic Stress

#### Modified Single Prolonged Stress

**FIGURE 1:**
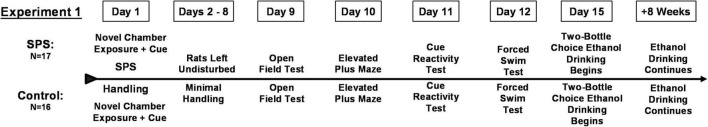
Timeline for Experiment 1.

Following acclimation to the facility, rats went through a modified single-prolonged stress (SPS) procedure (*N* = 17) based on the methods of Liberzon et al. (1997); Toledano and Gisquet-Verrier (2014), and Enman et al. (2015) or control handling (*N* = 16); see Figure 1 for the experimental timeline. On experimental day 1, rats were exposed to a novel chamber for 10 min, during which an intermittent tone played (70–80 dB; average frequency 1,000 Hz, range 550–1,500 Hz) for the last 5 min. Rats in the SPS group were then placed into individual decapicone devices for 2 h. Immobilization stress was followed by a group swim of 6–8 rats (round swim tank: 42 cm tall × 55 cm diameter, water 23–25°C) for 20 min. Rats were dried and put back into the novel chamber for 10 min following the same protocol as above (i.e., tone present for last 5 min). Then rats were rendered unconscious with isoflurane. Rats were returned to home-cages, housed in pairs and left undisturbed for 7 days except for addition of food and water (experimental days 2–8). Control animals were exposed to the novel chambers twice for 10 min each and otherwise remained in home-cages in the experimental room according to the same time schedule as the SPS rats. Control rats were housed in pairs, minimally handled and weighed during the 7-day period that SPS rats were undisturbed.

### Behavioral Phenotyping

#### Open Field Test

After SPS or control handling was complete, animals were tested with the open field test on day 9 in order to assess anxiety-like behavior after traumatic-stress exposure or control handling. On day 9, rats were individually placed in the center of the open field arena (45 cm × 45 cm) and their behavior was video recorded for 10 min. The lighting in the center of the open field was approximately 75 lx and the corners were approximately 45 lx. Videos were scored by two investigators, one blind to experimental group, and the number of entries and amount of time spent in center which was defined as head and shoulders across the threshold of the center area (15 cm × 15 cm) were measured.

#### Elevated Plus Maze Testing

Elevated plus maze performance was tested on day 10 following SPS or control handling as a second measure of anxiety-like behavior. The apparatus was constructed of black plastic (San Diego Instruments). The dimensions of the closed arms of the maze were 48.3 cm × 10.2 cm × 50.8 cm (L × W × H), the open arms were 48.3 cm × 10.2 cm (L × W), and the maze was 35.6 cm off the ground. Light levels in the closed arms were approximately 50 lx and the open arms were approximately 150 lx. Rats were placed in the center of the maze and video recorded for 10 min. Videos were scored for time and entries into the closed and open arms which was defined by the head, shoulders, and front paw entering an arm.

#### Reactivity to Trauma-Associated Cues

Using cues paired during SPS or control handling, cue-reactivity responses were measured using methods similar to Toledano and Gisquet-Verrier (2014). On day 11, rats were placed into a chamber for 10 min and during the last 5 min, an intermittent tone (70–80 dB) was played following the same SPS and control handling procedures described above. Behaviors in the chamber were video recorded for 10 min. Videos were scored for freezing behavior which was defined as the absence of any movement except respiration; time spent freezing and the number of freezing episodes were measured during the two 5-min periods.

#### Forced Swim Test

Depression-like behaviors were measured using a one-trial forced swim test on day 12. The swim test occurred in glass cylinders (46 cm × 20 cm diameter) filled with 23–25°C water to a depth of 36 cm. Rats were placed in the swim tank for 5 min and their behaviors were video-recorded. Rats were removed from the water, dried, and returned to their home cages. Behavior was classified as immobile if the rat exhibited no additional activity other than that necessary to remain afloat, swimming if there was forward movement through the tank, and climbing if there was upward movements of the forepaws against the tank sides. For all behavioral phenotyping, videos were scored by two investigators, at least one blind to treatment group. Scores were averaged for the final data set.

### Two-Bottle Choice Intermittent Access to 20% Ethanol

After traumatic-stress (SPS) or non-stressed control handling and behavioral testing, rats had intermittent access to 20% ethanol using a two-bottle choice procedure (Wayner et al., 1972; Simms et al., 2008; Vasudeva et al., 2015). Beginning on day 13, rats were singly housed in standard rat cages with two drinking bottles secured to the wire top of the cage. On Mondays, Wednesdays, and Fridays, one bottle contained drinking water and the other contained 20% ethanol in drinking water for 24 h. On the remaining days, the two bottles contained drinking water. The bottles were weighed every 24 h and ethanol bottle presented in a counterbalanced way to avoid side preference. Ethanol was available in this manner for 24 sessions over 8 continuous weeks, beginning on day 15 after SPS. The amount of ethanol consumed during each 24-h session was calculated as follows: [ethanol fluid (g) consumed × 0.162]/kg body weight to account for the specific density of ethanol in a 20% ethanol solution (Fisher et al., 2017). In addition to total ethanol consumption, ethanol preference was calculated as [ethanol fluid intake (g)/(ethanol fluid intake (g) + water fluid intake (g)] *100 to indicate what percent of daily fluid intake was derived from the ethanol solution.

### Artificial Intelligence- Support Vector Machine

In order to predict which animals were vulnerable, resilient, or neutral to subsequent ethanol consumption following traumatic stress in a reliable and reproducible way, an artificial intelligence algorithm was developed. A support vector machine which is a supervised machine learning algorithm was chosen. The support vector machine was trained and tested to classify and predict which rats would be resilient, neither, or vulnerable for heightened ethanol consumption based on data from open field time in center and elevated plus maze number of open arm entries. Training and test data included the cohort of rats (*N* = 17) exposed to SPS described in Experiment 1. The variables used to complete this were open field time in center, elevated plus maze number of open arm entries, and average ethanol consumption during weeks 6–8 (sessions 16–24). These endpoints were chosen based on results from Pearson correlations and multiple linear regressions between behavioral endpoints and ethanol consumption (see below). All variables were converted to *z*-scores for algorithm development. Using the behavioral scores, the algorithm was trained to predict ethanol consumption subsets as resilient, neither, or vulnerable from the *K*-means cluster. Labeled data for the support vector machine came from the unsupervised *k*-means cluster and formed the 80% of training data and 20% of test data used to build the support vector machine. The individual animals were randomly selected for training or test groups by the algorithm and this is reproducible if using a set.seed(125) function. This allowed the training and test data to be randomly selected and it is reproducible. This trained machine learning algorithm was used in Experiment 2 (below) to identify resilient and vulnerable animals for the molecular experiments without requiring subsequent ethanol exposure for phenotyping. Training data from a cohort of control rats (*N* = 16) were collected, and the same methods as described above were applied to generate a second support vector machine algorithm to filter out animals with naturally higher ethanol consumption.

### Experiment 2: Investigation of Neuropeptide Y as a Factor Underlying Vulnerability or Resilience to Heightened Ethanol Consumption Following Traumatic Stress

**FIGURE 2:**
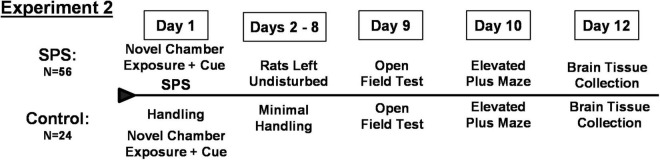
Timeline for Experiment 2.

Female rats in a second experiment underwent SPS (*N* = 56) or control handling (*N* = 24) followed by testing for anxiety-like behaviors on the open field test on day 9 and elevated plus maze on day 10 as described above for Experiment 1 (Figure 2). Values for time in center of the open field test and entries into the open arms of the elevated plus maze were analyzed by the algorithm developed from Experiment 1 in order to identify individual rats that classified as vulnerable or resilient to the traumatic stress and subsequent ethanol drinking. Values from control rats were analyzed by the algorithm developed in Experiment 1.

### Brain Tissue Collection

On day 12, 48 h after behavioral testing, rats were briefly exposed to CO_2_ and decapitated in an unconscious state. Brains were rapidly removed, immersed in isopentane cooled to −40°C for 30 s and stored at −80°C. Frozen brains were sectioned in a cryostat at −15°C into 300 μm sections. The BLA (interaural 6.24 mm), CeA (interaural 6.24 mm), and BNST (interaural 8.76 mm) were collected using 1 mm diameter punches taken bilaterally from two adjacent 300 μm sections according to the rat atlas of Paxinos and Watson (2007). Dissected brain samples were stored at −80°C until processed for RNA and protein isolation.

### RNA and Protein Extraction From Brain Tissue

Protein and RNA were extracted from frozen tissue samples using the *mir*Vana PARIS RNA and Native Protein Purification Kit according to the manufacturer’s instructions (#AM1556, Life Technologies, Carlsbad, CA, United States). All protein and gene expression assays were performed by an experimenter who was blinded to experimental group.

### qRT-PCR

RNA concentrations were measured with a NanoDrop 2000 spectrophotometer (Thermo Fisher Scientific). Before synthesizing cDNA with the High-Capacity cDNA Reverse Transcription Kit (Applied Biosystems, Waltham, MA, United States), RNA samples were diluted to the same RNA concentration. RT-PCR quantification was completed using TaqMAN Fast Advanced Master Mix and TaqMAN Gene Expression Assays (Thermo Fisher Scientific, Warrington, United Kingdom) for neuropeptide Y (Rn00561681_m1), neuropeptide Y Y1 receptor (Y1R, Rn02769337_s1), neuropeptide Y Y2 receptor (Y2R, Rn00576733_s1), and control 18s rRNA (Rn4319413E). The ^ΔΔ^ *C*t method was used to calculate relative fold change (Livak and Schmittgen, 2001) between controls, resilient and vulnerable rats.

### ELISA

Neuropeptide Y (NPY) levels were measured in individual brain regions using protein that was extracted by the miRVANA kit. Total protein was quantified using a BCA analysis (Rn23227, Thermo Fisher Scientific, Waltham, MA, United States). NPY was quantified by ELISA [S-1145, Peninsula Laboratories International, Inc. (San Carlos, CA, United States)]. Protein from each tissue sample was run in triplicate on 96-well microplates lined with neuropeptide Y selective polyclonal antibody specific for rats, together with NPY standards. Optical densities were measured using FluoroStar spectrophotometer (BMG Labtech, Ortenberg, Germany). NPY values in the samples were calculated by comparison to the NPY standard curve using linear regression analysis (GraphPad Prism).

### Data Analysis

Behavioral data were analyzed using an unpaired two-tailed *t*-test or one-way analysis of variance (ANOVA) with Bonferroni *post hoc* tests. Ethanol consumption across sessions was analyzed by two-way repeated measures ANOVA and Bonferroni post-tests. Pearson correlations and multiple linear regression were used in the analysis of the relationship between behavioral endpoints and ethanol consumption. Sub-populations were analyzed with *k*-means cluster and predictive categorization of resilient and vulnerable populations was completed by training a support vector machine (SVM). In the analysis of gene expression and protein levels, *a priori* exclusion criteria were set as >2 standard deviations from the means. Such samples were considered to be outliers and were removed from the data sets. This resulted in removal of 7 out of 279 values from qRT-PCR assay. GraphPad Prism 8 (La Jolla, CA, United States) was used for unpaired two-tailed *t*-test, ANOVAs, and Pearson correlation. SPSS (IBM, Armonk, NY, United States) was used for the multiple linear regression and *k*-means cluster. R version 3.5.2 (Vienna, Austria) and RStudio (RStudio Team, Boston, MA, United States) were implemented for the construction and use of the support vector machine.

## Results

### Experiment 1: Anxiety-Like Behaviors and Ethanol Drinking Following Single Prolonged Stress

**FIGURE 3:**
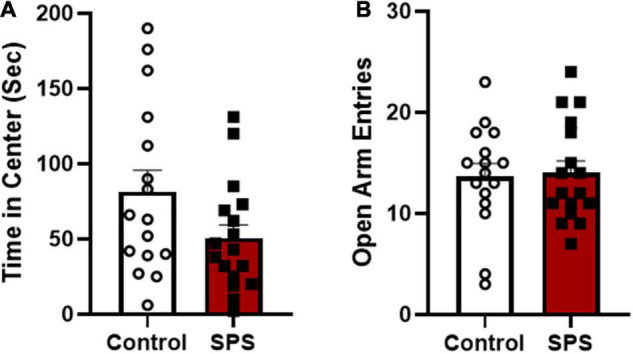
Anxiety-like behaviors were measured 8–9 days following SPS (*N* = 17) or control handling (*N* = 16). **(A)** Time spent (sec) in the center of the open field test is shown for control and SPS-exposed rats (control vs. SPS, *P* = 0.0718). **(B)** Number of open arm entries on the elevated plus maze testing is shown for SPS and non-stressed control rats (control vs. SPS, *P* > 0.05).

The first aim of this study was to determine if behavioral phenotyping following traumatic stress exposure in female rats could be used to predict individual vulnerability or resilience to ethanol drinking. The prediction was there would be inter-subject variability in anxiety-like behaviors and enhanced ethanol consumption after SPS exposure and that these two factors would be related. In experiment 1, SPS and control handled rats were tested on the open field test (day 9) and elevated plus maze (day 10) to assess anxiety-like behaviors. Analysis of group means revealed a trend toward less time in center of the open field for SPS rats compared to controls, however, this difference was not statistically significant [Figure 3A; two-tailed unpaired *t*-test: *t*(31) = 1.864, *P* = 0.0718]. Results from the elevated plus maze showed that there were no significant differences in open arm entries [Figure 3B; *t*(31) = 0.1782, *P* > 0.05], time in open arms [SPS: 140.0 s ± 18.05 vs. controls: 130.3 ± 15.29; mean + SEM *t*(31) = 0.4070, *P* > 0.05], or total number of arm entries [SPS rats: 32.65 ± 2.369 vs. controls: 30.75 ± 2.25 number of entries; *t*(31) = 0.5792, *P* > 0.05] between SPS and control groups. There were no significant differences between control and SPS rats in bouts of freezing to cues (SPS: context 46.82 ± 7.72, context + tone 127.06 ± 17.91 vs. control: context 46.69 ± 11.45, context + tone: 95.31 ± 15.41; mean + SEM) or immobility on the forced swim test (SPS: 31.91 ± 2.23 s vs. controls: 36.7 ± 2.47 s).

**FIGURE 4:**
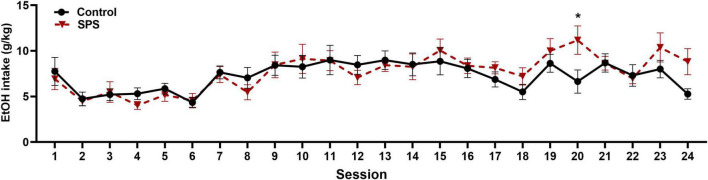
Ethanol consumption (g/kg/24 h session) for traumatic-stress exposed (SPS, *N* = 17) and non-stressed control rats (*N* = 16) is shown across 24 drinking sessions. There was a significant main effect of session (*P* < 0.0001), and a significant interaction between session and control/SPS main effects (*P* < 0.05). *Post hoc* tests revealed a significant difference in ethanol consumed between SPS and control rats during session 20 (*P < 0.05). Data are expressed as means + SEMs.

Following testing for anxiety-like behaviors, rats were provided access to ethanol in a two-bottle choice procedure. Twenty-four hour ethanol consumption was measured three times per week for 8 weeks for a total of 24 sessions commencing on day 15. Figure 4 shows mean ethanol consumption for SPS and control rats for each drinking session. Using a mixed-effect two-way ANOVA, there was a significant main effect of session [*F*(6,183) = 6.853, *P* < 0.0001], no significant main effect of experimental group [*F*(1,31) = 0.2637, *P* > 0.05], and a significant interaction [*F*(23,663) = 1.711, *P* = 0.0207]. *Post hoc* analys is revealed that SPS rats drank significantly more ethanol than controls on session 20 (*P* < 0.05).

### Anxiety-Like Behaviors in Single Prolonged Stress Population Identified to Predict Subsequent Ethanol Consumption

**FIGURE 5:**
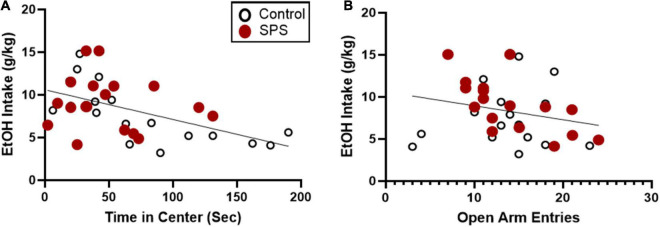
Correlation analyses between ethanol consumption during weeks 6–8 (sessions 16–24) and anxiety-like behaviors on the open field test and elevated plus maze. Both controls and SPS-exposed rats (*N* = 33) were included in the correlation analyses. **(A)** Time in center during open field testing was a significant indicator of ethanol consumption with a large effect size (**P = 0.0026, *r* = –0.5076). **(B)** Open arm entries during elevated plus maze testing was not significantly correlated with ethanol consumption (*P* > 0.05, *r* = –0.2431).

Analysis of group means for open field testing and elevated plus maze did not show significant differences between SPS and control-handled rats. However, there was high variability in anxiety-like behaviors and ethanol drinking suggesting individual differences in response to traumatic stress exposure. Subsequent analyses were performed to identify predictive factors linked to heightened ethanol consumption following traumatic stress exposure. In order to determine which measures were most predictive of ethanol consumption during weeks 6–8 when ethanol drinking was well-established, correlation analyses were performed. Individual control and SPS animals’ scores for open field time in center were strongly correlated with ethanol consumption in weeks 6–8, with a large effect size (Figure 5A; *P* = 0.0026, *r* = −0.5076, *N* = 33). Separate analyses for SPS and control groups reveal a significant correlation between time in center and ethanol consumption for the controls (*P* = 0.0034, *r* = −0.6848, *N* = 16) but not for SPS (*P* > 0.05, *r* = −0.1456, *N* = 17). Elevated plus maze number of open arm entries was not significantly correlated with ethanol consumption in weeks 6–8 when both SPS and control groups were considered together (Figure 5B; *P* > 0.05, *r* = −0.2431, *N* = 33). However, analysis of SPS data only reveal a significant correlation between open arm entries and ethanol consumption (*P* = 0.0041, *r* = −0.6579, *N* = 17); correlation analysis for the control group was not significant (*P* > 0.05, *r* = 0.123, *N* = 16). There was no significant correlation between any measures of cue reactivity or forced swim and ethanol drinking. These results indicate that the endpoint of time in center of the open field test was most predictive of subsequent ethanol consumption.

To determine if multiple behavioral variables after control handling or SPS were significantly related to ethanol drinking and would provide a better predictive model, a multiple linear regression was conducted. When used together, open field time in center and elevated plus maze number of open arm entries factors were significantly correlated to ethanol drinking during weeks 6–8 [*F*(2,32) = 8.93, *P* = 0.001]. The predictive power of these factors was moderately large based on the *R*^2^ = 0.37, adjusted *R*^2^ = 0.33 (Table 1). Other combinations including reactivity to trauma-associated cues and immobility on the forced swim test were assessed using correlation and multiple linear regression analysis, however, they were not significant predictors of ethanol consumption during weeks 6–8. When a multiple linear regression analysis was applied to anxiety-like endpoints of open field time in center and elevated plus maze number of open arm entries, these factors were most predictive of subsequent ethanol consumption with a moderate effect size. As such, these two variables were used to develop an algorithm to identify individuals that were resilient or vulnerable to SPS-induced anxiety and ethanol drinking.

**TABLE 1:** Results of correlation analyses of individual responses to traumatic-stress exposure characterized by open field test time in center and elevated plus maze number of open arm entries predicting subsequent ethanol consumption during weeks 6–8.

Predictors	Equation factors					Correlation	
	*B*	β	*SE*	*t*	*P*	Bivariate	Partial
Open field test	−0.006	−0.619	0.018	−3.801	0.001	−0.147	−0.010
Elevated plus maze	−0.051	−0.555	0.135	−3.407	0.025	−0.658	−0.641

### *K*-Means Cluster Unsupervised Machine Learning Algorithm Identified Three Subpopulations Within the SPS Group Based on Anxiety-Like Behavior and Subsequent Ethanol Consumption

To determine if different populations existed within the SPS group (*N* = 17), an unsupervised machine learning algorithm, *k*-means cluster was completed to identify subpopulations in an unbiased way. The machine learning algorithm was implemented using SPSS software (IBM, Armonk, NY, United States). *K*-means is a non-hierarchical clustering analysis that separates subpopulations (Forgy, 1965; Hartigan and Wong, 1979; Nanetti et al., 2009). Open field time in center (sec), elevated plus maze number of open arm entries, and average ethanol consumption during weeks 6–8 (gm/kg body weight) were converted into *z*-scores for this analysis to ensure equitable comparison.

**FIGURE 6:**
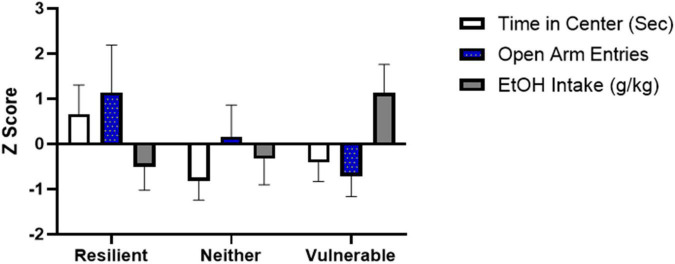
A 3 cluster k-means analysis for resilient (*N* = 4), neither (*N* = 6), and vulnerable (*N* = 7) groups. Mean + SEM *Z*-scores for open field time in center, elevated plus maze open arm entries, and weeks 6–8 ethanol consumption are shown for the 3 clusters.

The population classification revealed the SPS groups clustered into 3 distinct subpopulations (Figure 6 and Table 2). One cluster, which was classified as the resilient group, had both low anxiety-like behavior and low ethanol consumption (*N* = 4), whereas another cluster, termed the vulnerable group, had high anxiety-like behavior and high ethanol consumption (*N* = 7). As seen in Figure 6 and Table 2, the *k*-means factors were significant for open field time in center [*F*(2,14) = 11.810, *P* = 0.001], elevated plus maze number of open arm entries [*F*(2,14) = 9.081, *P* = 0.003] and average ethanol consumption during weeks 6–8 [*F*(2,14) = 13.9987, *P* < 0.001]. A similar algorithm was generated for the rats exposed to control handling (*N* = 16) in order to determine if a subset of the control population was predisposed to an ethanol preference (data not shown, cluster *N* = 4). Results identified 3 rats that showed high anxiety on open field time in center and high ethanol consumption.

**TABLE 2:** Resilient, neither, and vulnerable *K*-means cluster for open field test (OFT) time in center, elevated plus maze (EPM) number of open arm entries, and ethanol consumption during weeks 6–8.

	Resilient (*N* = 4) Mean (*SD*)	Neither (*N* = 6) Mean (*SD*)	Vulnerable (*N* = 7) Mean (*SD*)	*F* pairwise contrasts
OFT	1.32 (0.88)	−0.70 (0.58)	−0.15 (0.57)	11.81*** 1 > 2***, 1 > 3**
EPM	1.11 (1.05)	0.14 (0.70)	−0.75 (0.45)	9.08** 1 > 3***
EtOH weeks 6–8	−0.75 (0.55)	−0.59 (0.60)	0.94 (0.66)	13.99*** 1 < 3***, 2 > 3***

**P < 0.01 and ***P < 0.001.

### *K*-Means Cluster Subpopulation Validation

**FIGURE 7:**
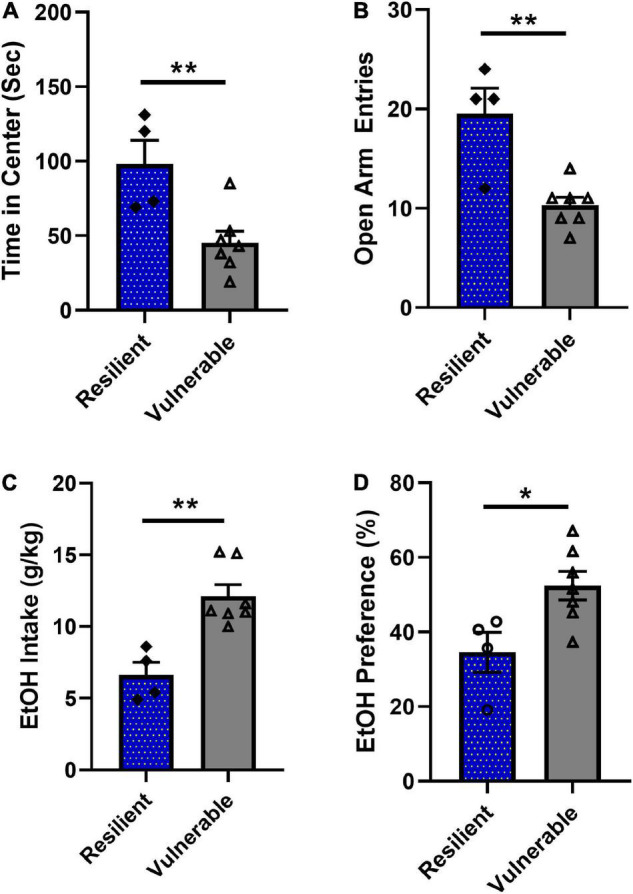
Behavioral phenotype of rats classified as resilient (*N* = 4) or vulnerable (*N* = 7). **(A)** Vulnerable rats spent significantly less time in center during the open field test compared to the resilient group (**P < 0.01). **(B)** Vulnerable rats had significantly lower numbers of open arm entries during elevated plus maze testing (**P < 0.01). **(C)** Vulnerable rats consumed significantly more ethanol than resilient rats during weeks 6–8 (**P < 0.01). **(D)** Vulnerable rats had significantly higher ethanol preference during weeks 6–8 compared to the resilient group (*P < 0.05). Data are expressed as means ± SEM and analyzed by two-tailed *t*-test.

In order to further demonstrate that the k-means cluster analysis identified different subpopulations of SPS rats, resilient and vulnerable groups were compared for anxiety-like behaviors and ethanol consumption during weeks 6–8. The vulnerable rats spent significantly less time in the open field center [Figure 7A, *t*(9) = 3.386, *P* = 0.008] and had significantly less elevated plus maze open arm entries [Figure 7B, *t*(9) = 4.197, *P* = 0.0023]. Analysis of mean ethanol consumed and mean ethanol preference during weeks 6–8 for each rat showed that vulnerable rats consumed significantly more ethanol than resilient rats [Figure 7C, *t*(9) = 4.197, *P* = 0.0023] and likewise, vulnerable rats had a greater preference for ethanol than resilient rats 6–8 [Figure 7D, *t*(9) = 2.759, *P* = 0.0221]. These results demonstrate that the unsupervised machine learning algorithm successfully identified two populations of rats, labeled as ‘resilient’ and ‘vulnerable,’ that were phenotypically different in terms of anxiety-like and ethanol drinking behaviors. As such, the algorithm could be used in Experiment 2 to predict which rats would be resilient or vulnerable to high ethanol consumption based on their anxiety scores alone following SPS exposure.

### Artificial Intelligence Algorithm Using a Supervised Machine Learning Support Vector Machine to Predict Resilient and Vulnerable Phenotypes

Using the subpopulations from the *k*-means cluster analysis, a support vector machine was programmed to predict resilient, neither, or vulnerable phenotypes based on anxiety-like behavior scores. A support vector machine is a supervised machine learning algorithm that can be trained to effectively predict outcomes with future data sets (Noble, 2006). The classification support vector machine used a cost function of 10 and a gamma function of 2 with a radial kernel. 80% of the data was used for training the algorithm and 20% was used to test the accuracy. The support vector machine was highly significant and accurately predicted vulnerable, neither, or resilient individuals 75% of the time using open field time in center and elevated plus maze number of open arm entries classifiers (confidence interval = 75–100%, *P* < 0.00001).

### Experiment 2: Neuropeptide Y System Regulation in Vulnerable and Resilient Subpopulations Following Single Prolonged Stress. Phenotype of Resilient and Vulnerable Populations Following Single Prolonged Stress

A separate cohort of female rats underwent SPS (*N* = 56) or control handling (*N* = 24), followed by assessment of anxiety-like behaviors on the open field test and elevated plus maze. Data on open field time in center and elevated plus maze number of open arm entries were subjected to analysis by the algorithm developed in Experiment 1 in order to identify individual rats predicted to be vulnerable or resilient to high ethanol drinking. Of the 56 rats that underwent SPS exposure, 10 were identified as vulnerable and 9 as resilient using the algorithm. The algorithm applied to the control group (*N* = 24) predicted 5 rats to be high drinkers (i.e., ‘vulnerable’), 13 rats to be low drinkers (i.e., ‘resilient’) and 6 rats as ‘neither.” Further analysis of *Z*-scores for open field time in center and elevated plus maze open arm entries was used to select the final control group (*N* = 12) which consisted of the 6 subjects from the ‘neither’ designation and 6 subjects from the ‘resilient’ designation with the lowest mean *Z*-scores from the two measures.

**FIGURE 8:**
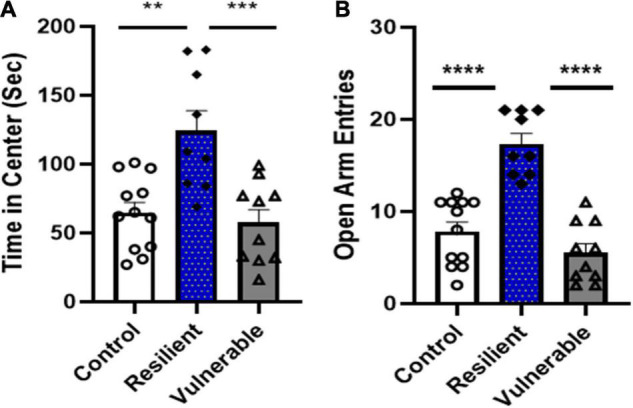
A separate cohort of rats were tested for anxiety-like behaviors on the open field test and elevated plus maze after control handling or SPS exposure. SPS rats were classified as vulnerable or resilient based on anxiety scores using the algorithm developed. **(A)** During open field, the resilient group spent significantly more time in center of the open field test than the vulnerable group (***P < 0.001) and control group (**P < 0.01). **(B)** The elevated plus maze test showed the resilient group had significantly higher open arm entries than the vulnerable (****P < 0.0001) or control (****P < 0.0001) groups. Data are expressed as means ± SEM; controls *N* = 12, resilient *N* = 9, vulnerable *N* = 10.

Analysis of behavioral scores for subjects identified as vulnerable, resilient or controls demonstrated group differences in anxiety-like phenotypes. A one-way ANOVA of open field time in center revealed a significant difference [*F*(2,28) = 11.52, *P* = 0.0002; Figure 8A]. Bonferroni *post hoc* tests showed the resilient group spent significantly more time in center than the control (*P* < 0.01) and vulnerable group (*P* = 0.0005); the control and vulnerable groups were not significantly different (*P* > 0.05). A one-way ANOVA of open arm entries on the elevated plus maze revealed a significant difference [*F*(2,28) = 31.66, *P* < 0.0001; Figure 8B]. Bonferroni *post hoc* tests showed the resilient group had significantly more open arm entries than the controls (*P* < 0.0001) and vulnerable group (*P* < 0.0001). Open arm entries for the control and vulnerable groups were not significantly different (*P* = 0.3680). Thus, rats identified as resilient versus vulnerable following SPS exposure had significant differences in anxiety-like behaviors.

### Neuropeptide Y, Y1R, and Y2R Levels in the Amygdala and Extended Amygdala of Resilient and Vulnerable Female Rats

**FIGURE 9:**
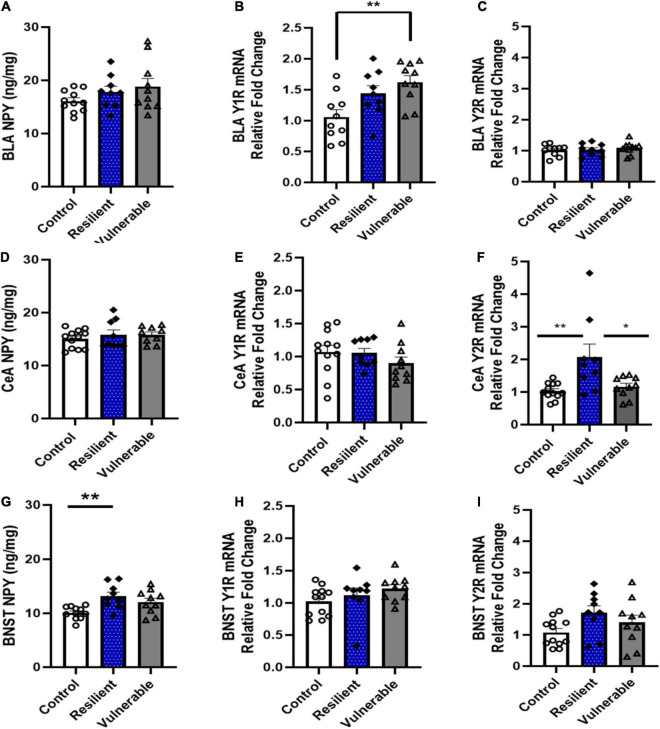
Levels of NPY protein, Y1R mRNA, and Y2R mRNA in the BLA, CeA and BNST in control and SPS-exposed rats classified as vulnerable or resilient. Traumatic stress exposure differentially effected the NPY pathway in resilient and vulnerable rats in a brain region-specific manner. **(A–C)** In the basolateral amygdala (BLA), Y1 mRNA was higher in vulnerable compared to the control group. **(D–F)** In the central nucleus of the amygdala (CeA), resilient rats had higher Y2 mRNA levels than control and vulnerable groups. **(G–I)** In the bed nucleus stria terminalis (BNST), resilient rats had higher levels of NPY protein than the control group. Data are expressed as means ± SEM; Controls *N* = 12; resilient *N* = 9; vulnerable *N* = 10. *Post hoc* tests: *P < 0.05, **P < 0.01.

Levels of NPY protein, and Y1R and Y2R mRNA were measured by ELISA and qRT-PCR, respectively, in three brain regions involved in stress responses and ethanol drinking, the BLA, CeA, and BNST. Brain regions were obtained 48 h following their final behavioral testing. Results are shown in Figure 9.

In the BLA, the levels of NPY were not significantly different between control, resilient and vulnerable groups [*F*(2,27) = 1.681, *P* > 0.05; Figure 9A]. There was a significant difference in Y1R mRNA expression [*F*(2,26) = 6.304, *P* = 0.0059; Figure 9B]. Bonferroni *post hoc* analysis revealed that the vulnerable group had significantly higher expression of Y1R mRNA than the control group in the BLA (*P* < 0.01); there were no significant differences between the control and resilient (*P* > 0.05) or the resilient and the vulnerable group (*P* > 0.05). A one-way ANOVA showed no difference between controls, resilient, or vulnerable rats in Y2R mRNA expression [*F*(2,26) = 0.7155, *P* > 0.05; Figure 9C].

In the CeA, a one-way ANOVA showed no significant differences in NPY levels between groups [*F*(2,27) = 0.5877, *P* > 0.05; Figure 9D]. Likewise, there were no differences in Y1R mRNA expression [*F*(2,27) = 2.019, *P* > 0.05; Figure 9E]. However, Y2R expression was significantly different between groups [*F*(2,27) = 6.979, *P* = 0.0036; Figure 9F]. Bonferroni *post hoc* analysis revealed that the resilient group had significantly higher Y2R mRNA than the control (*P* < 0.01) and vulnerable groups (*P* < 0.05).

In the BNST, a one-way ANOVA determined that NPY protein levels were significantly different between groups [*F*(2,27) = 6.497, *P* < 0.01; Figure 9G]. The resilient group had higher NPY levels compared to controls (*P* < 0.01); all other comparisons were not significantly different (*P* > 0.05). There was a significant difference in Y1R expression [*F*(2,27) = 3.543, *P* < 0.05; Figure 9H], however, Bonferroni *post hoc* test was not significant. There was no differences between controls, resilient, or vulnerable rats in Y2R mRNA in the BNST [*F*(2,28) = 2.644 *P* > 0.05; Figure 9I]. Taken together, these data indicate changes in the NPY system following SPS occurred in a brain region-specific manner and were dependent on behavioral phenotype.

## Discussion

The current study investigated individual responses to traumatic stress exposure and a potential neurochemical mechanism underlying these differences. Individual differences in response to traumatic stress were hypothesized to influence subsequent ethanol consumption. A novel artificial intelligence algorithm was trained to predict resilient, neither and vulnerable phenotypes for ethanol drinking following SPS based on anxiety-like behaviors. Using this algorithm, resilient and vulnerable subpopulations were found to coexist within rats exposed to SPS. The experimental subjects used in this study were young adult female rats. Females were chosen based on the clinical literature which reports a higher incidence of PTSD and co-occurring alcohol use disorder in women than men when corrected for the level of trauma exposure (Kessler et al., 1995), and because of the dearth of information on females from preclinical studies of traumatic stress and ethanol.

Using the SPS rat model of PTSD, female rats were exposed to the stressors and their behaviors characterized by four tests that evaluated anxiety- and depression-like responses, and reactivity to stress-related cues: open field test, elevated plus maze, forced swim test, and cue-reactivity. Following behavioral phenotyping, ethanol consumption using the intermittent ethanol two-bottle choice method, was measured for 8 weeks. These procedures were selected to model the transition from traumatic stress exposure to alcohol use disorder seen in clinical populations. Using this model, the study was designed to create a method to predict individual differences in ethanol preference following traumatic stress in order to investigate the neurobiological underpinnings driving enhanced drinking behavior. A limitation to most preclinical studies using PTSD models has been the failure to consider individual responses to the stressor and how these individual responses influence neuroplasticity and subsequent behavior, including alcohol-seeking. The approach developed in this study provides a methodological advance to the investigation of co-occurring stress disorders and ethanol drinking. Similar to humans, only a subpopulation of experimental animals develops symptoms akin to PTSD (Toledano and Gisquet-Verrier, 2014) and heavy alcohol ingestion following exposure to traumatic stress. Combining stress-exposed experimental subjects with and without the desired phenotype (in this case, heightened anxiety and ethanol preference) could impact the validity of subsequent analysis investigating the biological underpinnings of these disorders.

To investigate potential molecular mechanisms of heightened ethanol drinking following traumatic stress, it was predicted that the NPY pathway would be altered following stress exposure in a way consistent with vulnerability to the traumatic stress and ethanol drinking. The study focused its investigation of the NPY system in areas of the amygdala and extended amygdala that are known to be involved in fear response, anxiety-like behaviors, and ethanol consumption. The BLA, CeA, and BNST are nuclei in the amygdala and the extended amygdala (Tye et al., 2011; Jennings et al., 2013). The BLA receives sensory information from thalamic and cortical areas about the perception of fear stimuli. The CeA integrates sensory information from the BLA and other areas, then sends output projections to multiple regions that drive behavioral responses to fear stimuli. The BNST, an area that receives BLA projections and is reciprocally connected with the CeA, is responsible for the development of anxiety states and sustained fear (Pape and Pare, 2010; Rodríguez-Sierra et al., 2016). They play key roles in fear learning, and dysregulated fear learning is a hallmark of PTSD (Careaga et al., 2016). NPY, Y1R, and Y2R are expressed in the amygdala and extended amygdala (Tasan et al., 2016; Wood et al., 2016; Mackay et al., 2019). In general, activation of postsynaptic Y1R reduces anxiety, whereas activation of presynaptic Y2R increases anxiety-like behaviors (Sørensen et al., 2004; Bowers et al., 2012; Reichmann and Holzer, 2016). NPY administration or overexpression is anxiolytic in several rodent models, including in the elevated plus maze and open field tests (Heilig et al., 1989; Broqua et al., 1995; Sørensen et al., 2004; Kornhuber and Zoicas, 2021), and this is mediated primarily through activation of Y1R (Bowers et al., 2012). The findings presented herein demonstrate the NPY pathway is differentially regulated in areas of the amygdala and extended amygdala of vulnerable and resilient populations. Vulnerable rats had higher levels of Y1R mRNA in the BLA compared with controls. Resilient rats had significantly higher Y2R expression in the CeA and NPY levels in the BNST than non-stressed controls.

In the BLA, NPY neurotransmission has been shown to play a role in modulating anxiety and fear-related behaviors (Sajdyk et al., 2002; Tasan et al., 2010). When NPY is directly injected into the BLA, animals show resilience to restraint stress and display greater social behaviors compared to vehicle-injected shams (Sajdyk et al., 2008). Likewise, intra-amygdala NPY attenuates conditioned fear expression (Fendt et al., 2009) and enhances fear extinction, whereas Y1R antagonist inhibits fear extinction (Gutman et al., 2008). In contrast, genetic knockdown of Y2R in the BLA decreases anxiety (Tasan et al., 2010). These findings support the contention that Y1R decrease and Y2R increase anxiety states and the regulation occurs, at least in part, at the level of the BLA. Our results indicate that vulnerable rats were more anxious and had higher levels of Y1R mRNA in the BLA than controls but had similar levels of NPY. Our results are in contrast to those Cui et al. (2008) who report increased NPY-immunoreactivity in neurons in the BLA 7 days following SPS in male rats. The rats were not behaviorally phenotyped as vulnerable or resilient which may contribute to the differences in the findings; our rats were at the two extremes in terms of behavioral responses to SPS. There also may be differences between males and females in the regulation of NPY following traumatic stress. Males and females need to be investigated side-by-side to establish important sex differences in the biological responses to traumatic stress.

The CeA receives sensory inputs from the BLA and cortex, and in turn provides behavioral output through the BNST. The BNST has reciprocal connections with the CeA that are important in anxiety responses and ethanol consumption (Gilpin et al., 2015). As a central component to this circuit, the CeA is critical for processing responses to traumatic stress and regulating anxiety valence and alcohol drinking (Gilpin et al., 2015). The results presented herein indicate that resilient rats had higher levels of Y2R mRNA in the CeA than vulnerable rats. Elevated Y2R in resilient rats may result in lower fear responses in agreement with the findings of Verma et al. (2015) who demonstrated that over-expression of a Y2R agonist, NPY3-36, specifically in the CeA diminishes acquisition and recall during cued fear conditioning. The finding of lower Y2R mRNA in the vulnerable group may indicate a down-regulation of presynaptic control of local GABA interneurons or reduced Y2R expression by CeA projection neurons. Prior work shows that genetic knockdown of Y2R mRNA in the CeA results in down-regulation of Y2R binding within the CeA, but also in the BNST, nucleus accumbens shell, and locus coeruleus (Tasan et al., 2010). Reductions of Y2R expression by CeA GABAergic interneurons could result in disinhibition of GABAergic projection neurons from the CeA to the BNST, thus potentially reducing anxiety and ethanol consumption in the resilient group (Pati et al., 2020). This possibility needs to be further explored. Although there is a paucity of prior studies that have investigated the regulation of Y2R following SPS or other traumatic stress models, it has been reported that SPS results in a reduction of Y2R mRNA in the locus coeruleus of male rats compared with controls (Sabban et al., 2018); Y2R mRNA in the amygdala was not measured in that study.

The BNST has afferent and efferent connections with the CeA, which are critically involved in the subjective feeling of anxiety and linked to alcohol consumption (Gilpin, 2012; Avery et al., 2016). Results from the present study show that NPY levels were higher in the BNST in the SPS-exposed resilient subpopulation compared to non-stressed controls. This is consistent with previous work showing that NPY, stress, and ethanol consumption are related. Higher NPY levels in the BNST are associated with increased adaptive coping in response to swim stress following chronic variable stressors (Hawley et al., 2010). NPY signaling through Y1R in the BNST reduces ethanol binge drinking (Pleil et al., 2015) and central administration of NPY suppresses alcohol seeking after stress exposure (Cippitelli et al., 2010). High drinking in the dark (HDID-1) mice have significantly lower NPY levels in the BNST compared to heterogeneous stock mice (Barkley-Levenson et al., 2016). Thus, NPY expression in the BNST is inversely related to stress responses including anxiety and ethanol consumption. In the present study, the higher levels of NPY in the BNST of resilient rats may be neuroprotective against harmful stressful stimuli and prevent increased anxiety and ethanol consumption.

Sex differences in tests of anxiety-like behaviors have been reported. Male and female rats show different behavioral responses to aversive or threatening stimuli; female rats show more active responding and higher locomotion while males express behavioral inhibition (Albonetti and Farabollini, 1995; Fernandes et al., 1999; Scholl et al., 2019). These divergent responses appear during testing on the elevated plus maze and warrant caution in interpretation. Thus, high activity of females on the elevated plus maze may reflect their active coping strategy and not reduced anxiety-like behavior (Fernandes et al., 1999). This is similar to responses in fear conditioning paradigms where females are more likely to show ‘darting’ behaviors rather than freezing (Gruene et al., 2015), but may differ from open field testing where sex differences are not always apparent (Scholl et al., 2019). Sex differences in ethanol consumption during two bottle choice also occur, with female rats drinking more ethanol than male rats (Priddy et al., 2017). Further, different responses to SPS and other models of traumatic stress have been reported between male and female rodents (Keller et al., 2015; Pooley et al., 2018; Albrechet-Souza et al., 2020).

The results presented here support prior work demonstrating that high anxiety is predictive of ethanol consumption, and that a positive relationship exists between anxiety-like behaviors and ethanol intake in females (Izídio and Ramos, 2007; Ornelas et al., 2021). The present study revealed that the phenotype after SPS that was most predictive of high ethanol drinking in females was anxiety scores on the elevated plus maze and open field test. Some studies, although not all, indicate that anxiety is predictive of ethanol consumption also in male rodents (Izídio and Ramos, 2007; Bahi, 2013; Barchiesi et al., 2021; Makhijani et al., 2021). Other work has shown that female rats are more likely to drink during social isolation which is anxiogenic and males are more likely to drink in groups, demonstrating anxiety is a more important driver for female ethanol consumption than for males (Varlinskaya et al., 2015). Thus, it is possible that different phenotypes may be more predictive of ethanol consumption in males and this needs further investigation. The NPY pathway also shows sex-dependent regulation in response to various types of stressors (Forbes et al., 2012; Karisetty et al., 2017). These studies suggest there are inherent differences in male and female responses to stress, including expression of anxiety-like behaviors, ethanol consumption and NPY pathway regulation. Future investigations measuring the predictive factors for later ethanol consumption in males is needed.

In summary, exposure to traumatic stress produces a range of responses in both animal and human populations. It is important to study individual responses to stress exposure in order to elucidate molecular mechanisms responsible for resilience and vulnerability, as well as to consider effective prevention and treatment approaches. This study developed and applied an artificial intelligence algorithm to identify individual differences following exposure to traumatic stress in female rats that enabled prediction of resilience or vulnerability to subsequent ethanol consumption. Investigation of the NPY pathway revealed that resilient animals had significantly higher levels of NPY in the BNST and higher expression of Y2R in the CeA. This suggests that enhanced NPY transmission in areas of the amygdala and extended amygdala may be important for resilience to traumatic stress exposure and in preventing high ethanol consumption in females. Artificial intelligence algorithms could be developed to detect and predict individual differences in humans exposed to traumatic stress in order to apply a therapeutic intervention that shifts vulnerable individuals to a resilient phenotype.

## Data Availability Statement

The raw data supporting the conclusions of this article will be made available by the authors, without undue reservation, to any qualified researcher.

## Ethics Statement

The animal study was reviewed and approved by Temple University Institutional Animal Care and Use Committee.

## Author Contributions

RD and EU: conceptualization, methodology, and writing – original draft. EU and TE: resources, supervision, and funding acquisition. RD, KC, MG, JM, and EU: investigation. DY: statistical consulting. RD, KC, MG, JM, DY, TE, and EU: writing – review and editing. All authors contributed to the article and approved the submitted version.

## Conflict of Interest

The authors declare that the research was conducted in the absence of any commercial or financial relationships that could be construed as a potential conflict of interest.

## Publisher’s Note

All claims expressed in this article are solely those of the authors and do not necessarily represent those of their affiliated organizations, or those of the publisher, the editors and the reviewers. Any product that may be evaluated in this article, or claim that may be made by its manufacturer, is not guaranteed or endorsed by the publisher.
